# Outcomes of endoscopic ultrasound as a one‐off pancreatic cancer screening tool for 122 high‐ and moderate‐risk patients

**DOI:** 10.1002/jgh3.12432

**Published:** 2020-10-17

**Authors:** Marios Efthymiou, Sujievvan Chandran, Leonardo Zorron Cheng Tao Pu, Allison Collins, Anton Rajadurai, Mehrdad Nikfarjam, Rhys Vaughan

**Affiliations:** ^1^ Endoscopy Unit, Department of Gastroenterology and Hepatology Austin Health Melbourne Victoria Australia; ^2^ University of Melbourne Parkville Melbourne Victoria Australia; ^3^ Institute for Breathing and Sleep Austin Health Melbourne Victoria Australia; ^4^ Department of Hepatobiliary Surgery Austin Health Melbourne Victoria Australia

**Keywords:** endoscopic ultrasound, pancreatic cancer, screening

## Abstract

**Background and Aim:**

Pancreatic cancer (PC) carries a poor prognosis and is often detected at later stages. Screening programs for moderate‐ and high‐risk people are still under debate. We present the results from a prospective study on endoscopic ultrasound (EUS) as a one‐off screening tool for pancreatic cancer screening.

**Methods:**

Asymptomatic patients with moderate‐ or high‐risk of PC were invited to participate. Moderate risk consisted of one first‐degree and at least one second‐degree relative with PC and no PC‐associated genetic mutations. High risk consisted of >1 first‐degree relatives with PC or PC‐associated mutations (i.e. BRCA2, Lynch Syndrome, Familial Atypical Multiple Mole Melanoma Syndrome, STK11, or PALB2). All included patients had genetic counseling and a screening EUS done. Primary outcome was the detection of PC on EUS. Secondary outcomes assessed the evolution of psychological symptoms based on the Impact of Events Scale (IES) and Personal Consequences Questionnaire (PCQ) before and after the screening took place.

**Results:**

A total of 122 patients had a screening EUS performed between 2013 and 2019; 60 were male, 55.8 years was the mean age, 78 were at high risk for PC, and 25 had PC‐associated mutations. No pancreatic cancers were identified at the one‐off EUS screening. Overall, patients' IES/PCQ scores did not change after screening and feedback of no malignancy, with the exception of females (less concerned about PC after screening EUS).

**Conclusions:**

EUS did not detect any PCs in either a moderate‐ or high‐risk population as a one‐off screening method. The EUS procedure and genetic counseling improved psychological symptoms for the female subset of this population.

## Graphical abstract

There is still paucity of data on screening for pancreatic cancer. Although medical consensus seems to advocate for screening through endoscopic ultrasound or computer tomography/magnetic resonance cholangiopancreatography for “high‐risk” individuals, more data are needed to determine what the benefits are of such screening. Our study presents data on a cohort of individuals at moderate and high risk for pancreatic cancer.

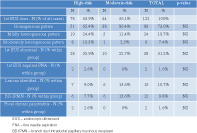

## Introduction

In 2019, Australia registered over 3500 new pancreatic cancer (PC) cases (11.6 per 100 000) and more than 3000 PC deaths (11.6 per 100 000).^1^ Mortality rates for PC have not significantly changed for decades. Most patients with symptomatic PC have advanced and/or metastatic disease at presentation. The 5‐year survival rate has been reported not to surpass 9%.^2^, ^3^ As in many cancers, early diagnosis leads to improved prognosis and forms the basis of screening programs.

Both family history of PC and PC‐associated genetic mutations have been shown to contribute to elevating the risk for PC.^4^ Approximately 5–10% of PCs are due to genetic susceptibility and/or familial aggregation. Familial aggregation of PC in some families is caused by germline mutations in known cancer predisposition genes.^5^ So far, four distinct genetic syndromes have been identified to greatly increase the risk of PC. The first is Peutz‐Jeghers Syndrome, which is caused by germline mutations in the STK11/LKB1 tumor suppressor gene [relative risk (RR) = 132, cumulative risk from age 15 to 64 years = 36%]^6^; the BRCA2 gene mutation (10‐fold higher risk for PC than the general population)^7^, ^8^, ^9^; the CDKN2A gene mutation associated with the Familial Atypical Multiple Mole Melanoma (FAMMM) Syndrome (16% lifetime risk of PC)^10^; and hereditary pancreatitis mostly associated with the SPINK1 gene mutation (cumulative risk of PC of 8–11% at the age of 50 years).^11^

Unfortunately, the genetic basis for most of the cases of familial PC is not known. Nonetheless, the risk of PC in these kindreds has been estimated. Prospective analysis of incident PCs in the Johns Hopkins National Familial Pancreas Tumor Registry kindreds, performed as part of the Johns Hopkins GI SPORE study, demonstrated that the risk of PC in persons with >2 affected family members is high (RR = 9.0, 95% confidence interval [CI] = 4.5–6.1). In individuals with three affected first‐degree relatives (FDRs), the risk for PC is elevated 32‐fold. In those with two affected first‐degree relatives, the estimated risk is 6.4.^12^

There are three known precursor lesions to PC: intraductal papillary mucinous neoplasm (IPMN), mucinous cystic neoplasia (MCN), and pancreatic intraepithelial neoplasia (PanIN). PanIN is by far the most common lesion, and three grades of PanIN have been described as cellular atypia progresses from low‐grade to high‐grade dysplasia. Molecular studies revealed that PanIN‐2 and PanIN‐3 lesions represent a distinct step toward invasive carcinoma. While main duct IPMN and large MCN can be detected by computer tomography (CT) and magnetic resonance imaging (MRI), small MCN, branch duct IPMN, and PanIN can be detected only by endoscopic ultrasound (EUS). Importantly, IPMN and PanIN create a distinctive form of lobulocentric parenchymal atrophy that is detectable only by EUS.^13^ Currently, CT and magnetic resonance cholangiopancreatography (MRCP) remain the standard‐of‐care modality to identify PCs, while EUS is the best modality to stage PCs (sensitivity of 84% and a specificity of 97%)^14^, ^15^, ^16^ and diagnose small and early pancreatic neoplasia.

Currently, routine PC screening is not recommended in the general population.^17^ However, data suggest that screening of groups at a high risk of developing PC might be of benefit. The optimal approach to screening for early pancreatic neoplasia has not yet been established, and each protocol is slightly different but many use EUS as part of the strategy. The potential for a PC screening program has been discussed in the CAPS 1, CAPS 2, and CAPS consensus papers. In the CAPS 1 study, 36 patients were screened using only EUS, and the diagnostic yield of screening was 5.3%.^18^ In the CAPS 2 study, a 10% diagnostic yield of screening for preinvasive malignant lesions was obtained using EUS and CT.^19^ The CAPS consensus highlights the potential of screening but still recommends it only in academic centers as part of research protocols.

The main aim of the study was to determine the frequency of detectable PCs in high‐risk and moderate‐risk patients.

## Methods

This was a prospective, single‐center, observational study. All patients who were identified in the Gastroenterology, Hepatobiliary Surgery, or Genetics outpatient clinics as meeting the eligibility criteria were invited to participate. In addition, information on the study was provided through the PanCare website (https://www.pancare.org.au/research/research-we-support/).

### *Inclusion criteria*

The inclusion criteria and correspondent PC risk groups were defined as follows:

Moderate‐risk Group:

1. Age > 40 years old (or 10 years younger than the age of youngest relative with PC) and <80 years old;
2. Member of a family with at least two blood relatives with a history of PC and have a first‐degree relationship (parent, sibling, or child) with only one of the relatives with pathologically proven PC or precursor lesion, such as a main duct IPMN (MD‐IPMN) or multifocal PanIN‐3.
3. No genetic mutations identified.

High‐risk Group:

1. Age > 30 years old and <80 years old and
2. Clinical diagnosis of Peutz‐Jeghers Syndrome or carrier of germline STK‐11 mutation.

OR

1. Age > 40 years old and <80 years old (or 10 years younger than the age of youngest relative with pancreatic cancer);
2. Patient is carrier of a known BRCA2, Lynch Syndrome, FAMMM Syndrome, or PALB2; and
3. There is > =1 PC in the family in a likely mutation carrier.

OR

1. Age > 40 years old (or 10 years younger than the age of youngest relative with PC) and <80 years old and
2. Member of a family with two or more first‐degree blood relatives with a history of PC and with at least one of the relatives with pathologically proven PC or precursor lesion such as an MD‐IPMN or multifocal PanIN‐3.

OR

1. Age > 40 years old and <80 years old (or 10 years younger than the age of youngest relative with PC) and
2. Previous diagnosis of hereditary pancreatitis or known SPINK1 mutation.

All persons with known genetic mutation(s) were required to have proof of mutation status.

### *Exclusion criteria*

Patients were excluded if they had any of the following:

1. personal history of PC or previous pancreatic surgery;
2. medical illnesses that increase the risk of endoscopy and possible surgery: unstable angina, severe congestive heart failure requiring daily medication, severe chronic obstructive pulmonary disease (COPD) GOLD Classification 4 (FEV1 < 30%), pulmonary hypertension, and untreated severe obstructive sleep apnea syndrome (AHI >30 and ESS > 10);
3. history of severe chronic kidney disease with an estimated glomerulofiltration rate (eGFR) < 30 mL/min, acute renal failure, cirrhosis of the liver, or chronic active hepatitis;
4. poor Karnosfky performance status of <60;
5. subjects not enrolled if they believed that they would not be interested in treatment of pancreatic abnormalities found during this study, such as possible pancreas surgery;
6. bleeding diathesis (clotting problems) or a history of thrombocytopenia (low platelet count);
7. previous gastric or biliary surgery other than cholecystectomy;
8. cancer (other than basal cell of the skin) within last 5 years, not in remission;
9. history of AIDS/HIV infection;
10. inability to provide informed consent;
11. pregnancy;
12. morbid obesity with body mass index >35; and
13. dementia

All eligible patients were referred to an initial EUS screening and had a genetic counseling consultation. Genetic mutation testing was performed at the discretion of the Austin Genetics Department. EUS procedures were performed by three experienced endosonographers (Rhys Vaughan, Marios Efthymiou, and Sujievvan Chandran, all with extensive EUS practice at an academic center). EUS was performed using linear‐array echoendoscopes (GF‐UCT180 series, Olympus Australia Corp, Notting Hill, Victoria, Australia) and ultrasound processors (Hitachi Aloka Medical, Ltd., Wallingford, CT, USA). The number and type of pancreatic lesions detected by EUS were recorded, as were the number of lesions that required fine‐needle aspiration (FNA). Genetic counseling was carried out for all eligible patients.

The primary outcome of this study was to assess the yield of EUS as a one‐off screening strategy to detect early PC. These were calculated per procedure/patient. Abnormal findings on EUS were considered only if lesions (e.g. nodules, cysts) were identified or if the pancreas was heterogeneous. Diagnoses such as fatty infiltration were considered variants of a normal EUS.

The secondary outcome on psychological symptoms was assessed through two questionnaires: Impact of Events Scale and Personal Consequences Questionnaire (Appendices I and II, respectively). These were conducted before and 1 month after counseling and EUS screening. Data were summarized as mean ± standard deviation (SD) or median (25th and 75th percentile) for continuous data and as frequency and percentages for categorical data. The scoring system for these questionnaires were considered continuous variables and measured as average score per questionnaire. The scoring system was based on previous research where the same four‐point scale was used.^20^ Each response was scored 0 for not at all, 1 for rarely, 3 for sometimes/some of the time, and 5 for often/quite a lot of the time. Questionnaires were considered “successfully responded” if at least half of the questions were answered. If not all (but at least half) questions were answered, the average was retrieved based on the responded questions only. In addition to these questionnaires, a one‐off question asked before the EUS had taken place was phrased as follows: “How worried are you that you may develop pancreatic cancer?,” and the patient could choose between five options (1 to 5), ranging from “not at all worried” (i.e. 1) to “extremely worried” (i.e. 5).

For the questionnaires' responses pre‐ and post‐EUS, scores were compared using the related‐samples Wilcoxon signed‐rank test based on the normality assumption. The Kolmogorov–Smirnov test was used to assess the data distribution. Independent categorical data were assessed with the Chi‐squared test and continuous variables with the *t* test. A *P* value of <0.05 was considered significant. Statistical analyses were performed with SPSS statistical software (IBM Corp. 2020. IBM SPSS Statistics, Version 26.0., Armonk, NY, USA).

This study was approved by the Austin Health Human Research Ethics Committee under reference number LNR/18/Austin/254. This Committee is constituted in accordance with the NHMRC National Statement on Ethical Conduct in Human Research (2007) and incorporates all updates.

## Results

As per the inclusion and exclusion criteria, a total of 122 patients consented and underwent screening EUS. The average age of participants was 59 years, and they was an equal number of males and females. The high‐risk group consisted of slightly older people, had a higher percentage of the male gender, and had a lower percentage of active smokers (Table 1).

**Table 1:** Cohort demographics

	High risk		Moderate risk		Total		*P*‐value
	Mean	SD	Mean	SD	Mean	SD	
Age (years)	57.4	10.6	53.0	9.6	55.8	10.5	0.02
BMI (kg/m^2^)	27.9	4.2	28.0	6.8	27.9	5.3	NS
	***n***	**%**	***n***	**%**	***n***	**%**	
Gender: Male	45	57.7	15	34.1	60	49.2	0.01
Mental health issue	25	32.5	11	25.0	36	29.8	NS
Born in Australia	58	74.4	32	72.7	90	73.8	NS
Caucasian	70	89.7	37	84.1	107	87.7	NS
0 FDR with PC	1	1.3	0	0.0	1	0.8	<0.001
1 FDR with PC	19	24.4	44	100.0	63	51.6	
2 FDR with PC	46	59.0	0	0.0	46	37.7	
3 or more FDR with PC	12	15.4	0	0.0	12	9.8	
0 SDR with PC	57	73.1	0	0.0	57	46.7	<0.001
1 SDR with PC	15	19.2	24	54.5	39	32.0	
2 SDR with PC	4	5.1	18	40.9	22	18.0	
3 or more SDR with PC	2	2.6	2	4.5	4	3.3	
Tested for mutation	27	34.6	2	4.5	29	23.8	<0.001
Peutz‐Jeghers	1	1.3	0	0.0	1	0.8	<0.001
PALB2^†^	4	5.1	0	0.0	4	3.3	
BRCA2	12	15.4	0	0.0	12	9.8	
CDKN2A	8	10.3	0	0.0	8	6.6	
Current smoker	3	3.8	6	13.6	9	7.4	0.047
Former smoker	24	30.8	18	40.9	42	34.4	NS
Regular alcohol intake	53	67.9	32	72.7	85	69.7	NS

^†^ One patient had VUS for PALB2.

BMI, body mass index; FDR, first‐degree relative; PC, pancreatic cancer; SDR, second‐degree relative.

There were no statistically significant differences between the moderate‐ and high‐risk groups regarding EUS findings, either regarding specific findings (e.g. number of branch duct [BD]‐IPMN) or more generic abnormalities (e.g. heterogeneity pattern). However, only patients in the high‐risk group required FNA for further clarification of suspicious lesions. Both FNAs came back negative for malignancy. Details on EUS findings can be found in Table 2. There were no PCs found in any of the initial screening EUS of this cohort.

**Table 2:** Endoscopic ultrasound (EUS) findings

	High risk		Moderate risk		Total		*P*‐value
	*n*	%	*n*	%	*n*	%	
First EUS done—*n* (% of all cases)	78	63.9	44	36.1	122	100	
Homogeneous pattern	51	65.4	38	86.4	89	73.0	NS
Mildly heterogeneous pattern	19	24.4	5	11.4	24	19.7	NS
Moderately heterogeneous pattern	8	10.3	1	2.3	9	7.4	NS
First EUS abnormal—*n* (% within group)	28	35.9	10	22.7	38	31.2	NS
First EUS required FNA—*n* (% within group)	2	2.6	0	0	2	1.6	NS
Lesions identified—*n* (% within group)	7	9.0	6	13.6	13	10.7	NS
BD‐IPMN—*n* (% within group)	6	7.7	6	13.6	12	9.8	NS
Focal chronic pancreatitis—*n* (% within group)	2	2.6	0	0	2	1.6	NS

BD‐IPMN; branch duct intraductal papillary mucinous neoplasm; EUS, endoscopic ultrasound; FNA, fine‐needle aspiration.

For the final analysis regarding questionnaire responses, 111 successfully responded to the pre‐EUS questionnaires and 71 responded to the post‐EUS questionnaires. However, only 69 participants had matched questionnaires for comparison. The questionnaire results showed that the screening EUS/genetic counseling had no effect in mitigating or worsening psychological symptoms overall but did improve the psychological symptoms for the female subgroup. Details on the evolution of psychological symptoms scores pre‐ and postscreening can be found in Table 3.

**Table 3:** Psychological evaluation scores

	Moderate risk				High risk			
	*n*	Mean	SD	*P*‐value	*n*	Mean	SD	*P*‐value
IES pre	22	0.77	0.83	0.17	47	0.96	1.10	0.36
IES post		0.65	0.91			0.87	0.96	
PCQ pre		0.57	0.83	0.15		0.73	0.96	0.37
PCQ post		0.36	0.62			0.68	1.03	
	**Male**				**Female**			
IES pre	34	0.52	0.65	0.37	35	1.27	1.18	**<0.01**
IES post		0.63	0.85			0.96	1.01	
PCQ pre		0.32	0.50	0.39		1.02	1.10	**<0.01**
PCQ post		0.41	0.88			0.73	0.95	
	**≤56 years**				**>56 years**			
IES pre	32	0.85	1.04	0.41	37	0.95	1.01	0.19
IES post		0.77	0.92			0.81	0.98	
PCQ pre		0.64	0.93	0.20		0.71	0.92	0.39
PCQ post		0.56	0.97			0.59	0.90	

IES, impact of events scale; PCQ, personal consequences questionnaire.

## Discussion

PC screening has been proposed by several groups, and more recently in a consensus, as potentially beneficial but is still only carried out in academic tertiary centers.^17^ The effects as such have been studied in Australia with a variable yield and effect on psychological symptoms.^21^, ^22^ This study adds to this body of knowledge in the Australian setting.

In our cohort, with an initial screening EUS, we were unable to detect any PC, as opposed to a similar study from NSW, which found one case with a one‐off EUS screening (1.0% of the cohort).^21^ Although we did not identify a PC on an initial EUS, PC was diagnosed early on follow‐up EUS in one individual. This was carried out after a finding of marked lobularity on the initial EUS, which led to a follow‐up CT scan a couple of months later, and this did not identify any concerning pathology. The second EUS study, performed 1 year following the initial study, was incomplete due to poor tolerance of sedation. However, a slight increase in the caliber of the pancreatic duct was noted on this limited examination, and subsequent EUS and CT done in the following months confirmed PC early (<2 cm at the pancreatic body). The patient was treated with curative radical subtotal distal pancreatectomy and splenectomy. The specimen confirmed a pT2N0 pancreatic adenocarcinoma. This case was part of our high‐risk cohort (mutation of BRCA2, one FDR, and one second‐degree relative [SDR] with PC). This lends some support to a potential strategy of using one‐off EUS to stratify high‐risk patients who warrant more intensive surveillance, although this requires further study.

In our cohort of patients with high/moderate risk for PC, we did not identify a short‐term psychological benefit of a screening protocol involving genetic counseling and screening EUS. However, a beneficial effect on psychological symptoms was observed in the female subgroup. Our findings are consistent with those of another study from New South Wales, where the IES scores also improved in the female subgroup comparing pre‐ and postscreening (after 1 year) questionnaires.^22^ Interestingly, in contrast to our cohort, the NSW study did not detect a statistically significant benefit in psychological symptoms on the early questionnaire administered 1 month postscreening.

Our study presents findings on a relatively small cohort, and hence, there are limitations. Only 64% of our cohort were high‐risk patients, and future studies focusing only on this population are warranted. It is likely that the yield of such a strategy will be higher. This study looked at the effect of a one‐off EUS strategy to screen for PC and does not report on other modalities of screening or the outcomes of ongoing screening. These aspects should be investigated further in future research. Nevertheless, our results provide some input on the yield of a one‐off screening strategy and its immediate effect on patients' psychological symptoms.

In conclusion, EUS did not detect any PCs in either a moderate‐ or high‐risk population as a one‐off screening method. The EUS procedure and genetic counseling improved psychological symptoms for the female subset of this population. More studies are required to determine the ideal tool and target population for PC screening.

## Appendix I 1

### 1.1

Impact of events scale (IES)

Below is a list of comments made by people about being at risk for pancreatic cancer. Please tick a box to indicate how frequently these comments were true for you during *the last seven days*.

**Table:**

		Not at all	Rarely	Sometimes	Often
1	I thought about it when I didn't mean to	□	□	□	□
2	I avoided letting myself get upset when I thought about or was reminded of it	□	□	□	□
3	I tried to remove it from my memory	□	□	□	□
4	I had trouble falling asleep or staying.asleep because of pictures or thoughts about it that came into my mind	□	□	□	□
5	I had waves of strong feelings about it	□	□	□	□
6	I had dreams about it	□	□	□	□
7	I stayed away from reminders of it	□	□	□	□
8	I felt as if it wasn't real	□	□	□	□
9	I tried not to talk about it	□	□	□	□
10	Pictures popped up into my mind	□	□	□	□
11	Other things kept making me think about it	□	□	□	□
12	I was aware that I still had a lot of feelings about it, but I didn't deal with them	□	□	□	□
13	I tried not to think about it	□	□	□	□
14	Any reminder brought back feelings about it	□	□	□	□
15	My feelings were sort of numb	□	□	□	□

## Appendix II 1

### 1.1

Personal Consequences Questionnaire (PCQ)

We would like to know your experiences of the screening procedure and your thoughts and feelings about pancreatic cancer.

1. Over the *last week* have you experienced the following things because of thoughts and feelings about *pancreatic cancer*

**Table:**

		Not at all	Rarely	Some of the time	Quite a lot of the time
1	Had trouble sleeping	□	□	□	□
2	Experienced a change in appetite	□	□	□	□
3	Been unhappy or depressed	□	□	□	□
4	Been scared and panicky	□	□	□	□
5	Felt nervous or strung up	□	□	□	□
6	Felt under strain	□	□	□	□
7	Found you have been keeping things from those who are close to you	□	□	□	□
8	Found yourself taking things out on other people	□	□	□	□
9	Found yourself noticeable withdrawing from those who are close to you	□	□	□	□
10	Had difficulty doing things around the house which you normally do	□	□	□	□
11	Had difficulty meeting work or other commitments	□	□	□	□
12	Feeling worried about your future	□	□	□	□
